# Probing the pH Effect on Boehmite Particles in Water Using Vacuum Ultraviolet Single-Photon Ionization Mass Spectrometry

**DOI:** 10.3390/ijms26157254

**Published:** 2025-07-27

**Authors:** Xiao Sui, Bo Xu, Xiao-Ying Yu

**Affiliations:** 1 Materials Science and Technology Division, Oak Ridge National Laboratory, Oak Ridge, TN 37830, USA; xiaosui_1022@163.com; 2 College of Geography and Environment, Shandong Normal University, Jinan 250358, China; 3 Chemical Sciences Division, Lawrence Berkeley National Laboratory, Berkeley, CA 94720, USA; boxu2018@gmail.com

**Keywords:** boehmite, pH dependence, water cluster, SALVI, VUV SPI-MS, appearance energy (AE)

## Abstract

Boehmite has been widely used in theoretical research and industry, especially for hazardous material processing. For the liquid-phase treating process, the interfacial properties of boehmite are believed to be affected by pH conditions, which change its physicochemical behavior. However, molecular-level detection on cluster ions is challenging when using bulk approaches. Herein we employ in situ vacuum ultraviolet single-photon ionization mass spectrometry (VUV SPI-MS) coupled with a vacuum-compatible microreactor system for analysis at the liquid–vacuum interface (SALVI) to investigate the solute molecular composition of boehmite under different pH conditions for the first time. The mass spectral results show that more complex clustering of solute molecules exists at the solid–liquid (s–l) interface than conventionally perceived in a “simple” aqueous solution. Besides solute ions, such as boehmite molecules and fragments, the composition and appearance energies of these newly discovered solvated cluster ions are determined by VUV SPI-MS in different pH solutions. We offer new results for the pH-dependent effect of boehmite and provide insights into a more detailed solvation mechanism at the s–l interface. By comparing the key products under different pH conditions, fundamental understanding of boehmite dissolution is revealed to assist the engineering design of waste processing and storage solutions.

## 1. Introduction

As a multifunction nanofiller, boehmite (γ-AlOOH) has been widely used in industrial processes, such as polymer nanocomposites preparation, coating, and environmental treatment [1]. Boehmite is also a major component of high-level radioactive wastes because of its wide applications in hazardous material processing [2]. The fabrication, crystal structures, optical–electronic properties, particle morphology, and synthesis route of boehmite have generated extensive interests in theoretical simulations and engineering [3,4,5]. Recently, the interfacial properties of boehmite were found to have effects on its physicochemical behavior in a study of the liquid-phase treating process of sewage and waste sludge treatment [6]. However, most studies were conducted to characterize boehmite as a solid or in the liquid bulk phase, including scanning electron microscopy (SEM), [7] optical microscopy (OM) [8], transmission electron microscopy (TEM) [9], atomic force microscopy (AFM) [10], X-ray photoelectron spectroscopy (XPS) [11], and Fourier-transform IR spectroscopy [12]. The physicochemical properties at the liquid surface are different from that in the bulk because the liquid surface is not a perfect termination of the crystal structure [13]. Thus, better understanding of the structure of the boehmite–liquid surface is essential to improve the designs for waste processing and environmental solutions. Moreover, it is known that the pH value of waste sludge can affect both the composition and structure of the suspended solid particles [14]. Nevertheless, direct observations of the dissolution and solvation of γ-AlOOH, as well as the pH effect at the liquid surface, are scarce because it is challenging to probe the particle surface in the liquid phase directly.

Synchrotron-based single-photon ionization mass spectrometry (SPI-MS) with bright vacuum ultraviolet (VUV) photons, or VUV SPI-MS, is a powerful approach that has been used to investigate chemical systems and their evolution [15]. Compared with other ionization methods, such as charged particles or electron impact, VUV SPI-MS is a “soft” ionization method to detect complex systems with fewer fragments [16]. Moreover, appearance energies (AEs) acquired from photoionization efficiency (PIE) curves could improve the precision of chemical compound identification in accordance with known ionization energies (IEs) and mass-to-charge ratios (*m*/*z*) in the acquired mass spectrum.

Recent studies show that integrating the vacuum-compatible microreactor termed “system for analysis at the liquid–vacuum interface (SALVI)” and VUV SPI-MS makes it possible to study biological and environmental chemistry at the material interface involving liquid [15,17]. The liquid analysis is performed in high-vacuum conditions. Unlike SEM and time-of-flight secondary ion mass spectrometry (ToF-SIMS), tiny apertures a micrometer in diameter let liquid evaporate slowly and allow ionization above the aperture surface by the proton beam [18]. The liquid near the surface can compensate for the evaporation, and the temperature and composition in the main chamber will not change significantly [19]. Additionally, the bulk solute can diffuse to the surface and the concentration at the surface will not reduce along with evaporation [19]. Furthermore, the photoelectrons will be scattered by gas molecules during analysis, and the signal-to-noise ratios in the gas phase will be lower than those under vacuum [20]. Therefore, liquid analysis of equilibrated conditions in vacuum is possible and we have shown promising results using multiple platforms [21].

Herein, we present novel findings of the solid–liquid (s–l) interface of the dissolution and solvation of boehmite in different pH conditions. The hydration and hydroxyl reaction pathways of boehmite were investigated and expanded based on the newly detected species using VUV SPI-MS for the first time. Moreover, key products and reactants were first identified by mass spectral analysis and determination of appearance energies (AEs) of ions. The observation of boehmite-containing cluster peaks indicates that the solvated structures of boehmite depend on pH. Our results showed that more complex solute molecules exist at the s–l interface than conventionally perceived in liquid. These findings provide new understanding of chemical processes at the interfaces from boehmite solvation and dissolution in liquid.

## 2. Results and Discussion

### 2.1. VUV SPI-MS Spectral and AE Analysis of Boehmite Dissolution

**Figure 1:**
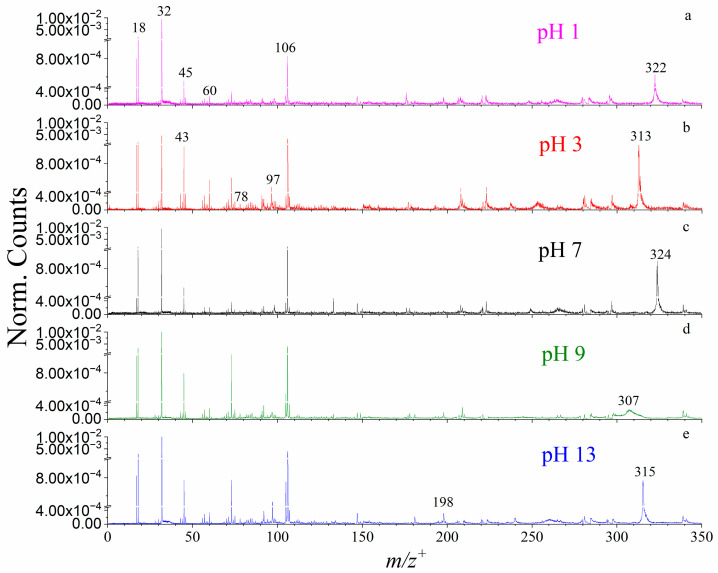
Normalized VUV SPI-MS spectral plots of boehmite at different pH observed at 12.5 eV: (**a**) pH 1, (**b**) pH 3, (**c**) pH 7, (**d**) pH 9, and (**e**) pH 13.

**Figure 2:**
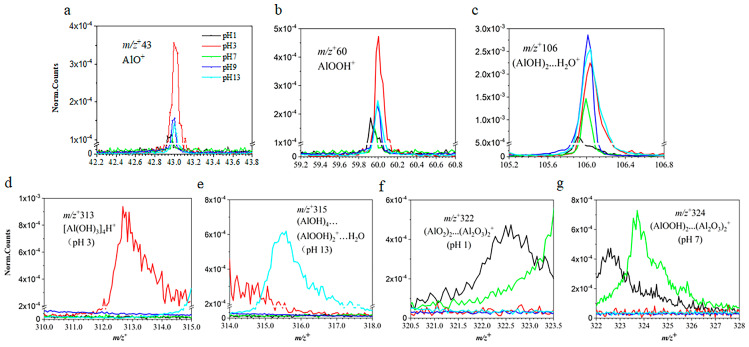
Normalized VUV-SPIMS spectral plots of boehmite at 12.5 eV showing key peaks: (**a**) *m*/*z*^+^ 43 AlO^+^, (**b**) *m*/*z*^+^ 60 AlOOH^+^, (**c**) *m*/*z*^+^ 106 Al(OH)_2_…H_2_O^+^, (**d**) *m*/*z*^+^ 313 [Al(OH)_3_]_4_H^+^, (**e**) *m*/*z*^+^ 315 (AlOH)_4_…(AlOOH)_2_^+^…H_2_O, (**f**) *m*/*z*^+^ 322 (AlO_2_)_2_…(Al_2_O_3_)_2_^+^, and (**g**) *m*/*z*^+^ 324 (AlOOH)_2_…(Al_2_O_3_)_2_^+^.

Representative VUV SPI-MS spectra were illustrated in Figure 1 and Figure 2. The findings suggest that boehmite dissolution in different pH conditions produce more than the known AlOOH solute in the molecular form. Furthermore, all AEs of the key products have been determined for the first time in this study, to our knowledge (Table 1). Specifically, several peaks related to boehmite, aluminum oxides, and hydroxides were identified from mass spectral analysis, such as *m*/*z*^+^ 43 (AlO^+^), *m*/*z*^+^ 60 (AlOOH^+^), and *m*/*z*^+^ 78 (Al(OH)_3_^+^). Their AEs were 10.8, 10.7 and 10.8 eV, respectively. In comparison, AlOOH and Al(OH)_3_ were also detected by static SIMS on the powder surface [22,23]. Additionally, the mass spectral results show peaks that were not reported before. For example, aluminum hydroxides with water clusters including *m*/*z*^+^ 97 (Al(OH)_2_^+^…2H_2_O, 11.2 eV) and *m*/*z*^+^ 106 ((AlOH)_2_^+^…H_2_O, 8.6 eV) were observed for the first time.

Another type of important dissolved ions observed in VUV SPI-MS spectra are cluster ions. These ions indicate ion pairs formed via weak intermolecular forces in the aqueous solution. For example, *m*/*z*^+^ 198 was identified as (AlOOH…Al(OH)_2_…Al(OH)_3_^+^, 10.3 eV) based on previous results of static SIMS analysis of solid boehmite [22,24]. These cluster ions have not been reported in liquid to the authors’ knowledge. *m*/*z*^+^ 313 was primarily assessed as ([Al(OH)_3_]_4_^+^, 11.2 eV). In addition, dissolved boehmite can form ion clusters like *m*/*z*^+^ 315 ((AlOH)_4_…(AlOOH)_2_^+^…H_2_O, 10.3 eV) and *m*/*z*^+^ 322 ((AlO_2_)_2_…(Al_2_O_3_)_2_^+^, 11.4 eV) in solutions in different pH conditions. The peak of *m*/*z*^+^ 324 was identified as ((AlOOH)_2_…(Al_2_O_3_)_2_^+^, 11.2 eV) based on the solute composition and mass matching. As shown in Figure 2d–g, the broad peaks consisting of the parent and protonated ions were likely caused by the proton-transfer process during VUV analysis [25]. It is worth noting that the PIE of these cluster ions exhibits similar features, implying that these newly detected cluster ions are possibly combined by semblable functional compounds, like aluminum oxides and aluminum hydroxides.

**Figure 3:**
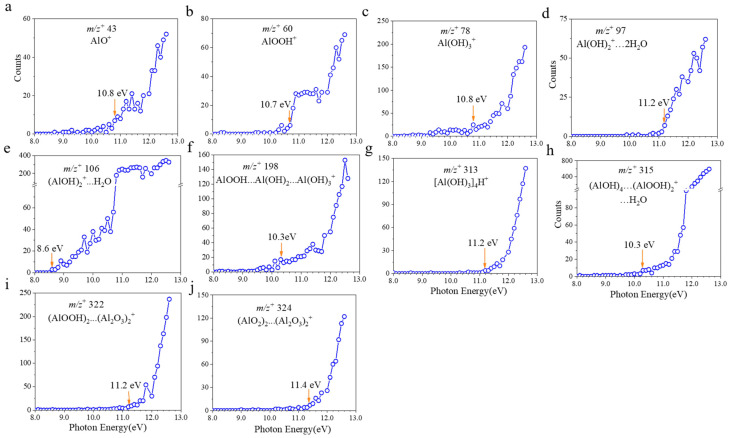
AEs of key observed ions of the dissolved boehmite particles using VUV SPI-MS: (**a**) *m*/*z*^+^ 43 AlO^+^, (**b**) *m*/*z*^+^ 60 AlOOH^+^, (**c**) *m*/*z*^+^ 78 Al(OH)_3_^+^, (**d**) *m*/*z*^+^ 97 Al(OH)_2_^+^…2H_2_O, (**e**) *m*/*z*^+^ 106 (AlOH)_2_^+^…H_2_O, (**f**) *m*/*z*^+^ 198 AlOOH…Al(OH)_2_…Al(OH)_3_^+^, (**g**) *m*/*z*^+^ 313 [Al(OH)_3_]_4_H^+^, (**h**) *m*/*z*^+^ 315 (AlOH)_4_…(AlOOH)_2_^+^…H_2_O, (**i**) *m*/*z*^+^ 322 (AlOOH)_2_…(Al_2_O_3_)_2_^+^, and (**j**) *m*/*z*^+^ 324 (AlO_2_)_2_…(Al_2_O_3_)_2_^+^.

Furthermore, AEs of boehmite, aluminum oxides, hydroxides, and cluster ions are presented in Figure 3. The red arrows indicate the values of the AEs for each detected key ion. These values, acquired from the VUV SPI-MS, are summarized in Table 1. Many cluster ions were first detected by VUV SPI-MS coupled with the SALVI microreactor. Besides hydration and hydroxyl configuration of boehmite particles, solvated ions and ion fragments can form more complex cluster ions in water as a solvent, presenting more insights into the fundamental process of solvation at the molecular level [2,23].

### 2.2. The pH Effects on Boehmite Dissolution

**Figure 4:**
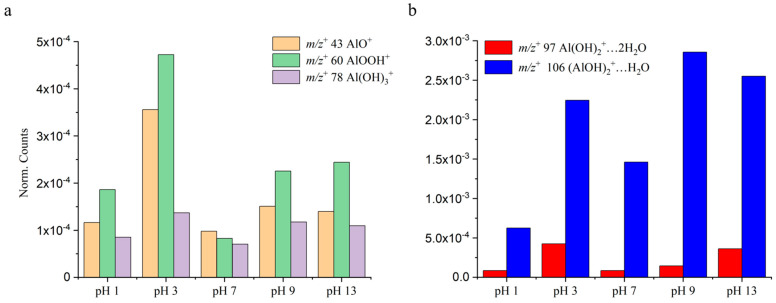
Comparison of pH dependence of representative boehmite AOH 60 components observed by VUV SPI-MS: (**a**) *m*/*z*^+^ 43 AlO^+^, *m*/*z*^+^ 60 AlOOH^+^, and *m*/*z*^+^ 78 Al(OH)_3_^+^, and (**b**) *m*/*z*^+^ 97 Al(OH)_2_^+^…2H_2_O and *m*/*z*^+^ 106 (AlOH)_2_^+^…H_2_O. Normalization is to total ion intensities.

The normalized intensities were used to compare key observed ions from the VUV SPI-MS spectra. The pH dependence of representative ions on boehmite dissolution is depicted in Figure 2. It is interesting to note that boehmite solvation products are detected with different intensities under different pH conditions. The new findings suggest that the dissolution of boehmite and solvation of particles in solvent actually produce more complex solvated ions and cluster ions with water, presenting a new understanding compared to the existing simpler picture of the presence of the molecular form of boehmite solvated in water. Specifically, the peaks of smaller aluminum oxides and hydroxides, for example, *m*/*z*^+^ 43 AlO^+^, *m*/*z*^+^ 60 AlOOH^+^, and *m*/*z*^+^ 78 Al(OH)_3_^+^, have the highest intensities in an acidic environment at pH 3. Also, alkaline conditions (i.e., pH 9, pH 13) can stimulate the formation of aluminum oxides and hydroxides, like AlOOH^+^ and Al(OH)_3_^+^ (Figure 4a). In contrast, these ions show lower normalized counts in the neutral solution at pH 7. Aluminum hydroxides with water clusters, including *m*/*z*^+^ 97 Al(OH)_2_^+^…2H_2_O and *m*/*z*^+^ 106 (AlOH)_2_^+^…H_2_O, revealed different features among the studied pH solutions. For example, for *m*/*z*^+^ 97 (Al(OH)_2_^+^…2H_2_O), its abundance in the acidic solution at pH 3 was slightly larger than in the strong alkaline solution at pH 13. As to *m*/*z*^+^ 106 (AlOH)_2_^+^…H_2_O, it showed higher intensities in alkaline solutions at pH 9 and 13. Also, the acidic condition of pH 3 can promote its formation.

Compared with aluminum oxides, aluminum hydroxides, and small cluster ions, the formation of larger cluster ions shows stronger dependence on pH conditions. As seen in Figure 1 and Figure S2, cluster ions have prominent presence at certain pH solutions. For instance, *m*/*z*^+^ 198 AlOOH…Al(OH)_2_…Al(OH)_3_^+^ was observed in alkaline conditions, namely pH 9 and 13; meanwhile, *m*/*z*^+^ 313 [Al(OH)_3_]_4_^+^ can only be detected in the acidic (pH 3) solution. Additionally, dissolved boehmite can form *m*/*z*^+^ 315 (AlOH)_4_…(AlOOH)_2_^+^…H_2_O in strong alkaline condition (pH 13), *m*/*z*^+^ 322 (AlO_2_)_2_…(Al_2_O_3_)_2_^+^ in strong acidic condition (pH 1), and *m*/*z*^+^ 324 (AlOOH)_2_…(Al_2_O_3_)_2_^+^ in neutral solution (pH 7). As summarized in Table 1, most cluster ions were detected at 12.5 eV for the first time. Cluster ions that contain boehmite and aluminum oxides tend to form in acidic solutions. In contrast, cluster ions that have boehmite hydroxides could be easily observed in alkaline solutions. As to the neutral pH 7 solution, boehmite, aluminum oxides, and hydroxides were detected; however, the detected species and intensities are fewer and their counts lower than those in acidic and alkaline conditions. Additional product identifications of boehmite are summarized in Table 1. Our results show that, in addition to the molecular form of boehmite, a variety of aluminum oxides, hydroxides, and cluster ions incorporating water molecules coexist in the solvent cage. Varying pH could influence the formation of hydrogen bonds, which will change the structure of cluster ions. Their relative abundances also vary depending on the pH condition, suggesting the hydronium ions and hydroxide ions actively participate in the dissolution process at the molecular level. Specifically, boehmite tends to form 1D nanorods or nanofibers in acidic conditions, whereas 2D and 3D nanostructures are detected in alkaline conditions [26]. In terms of composition, nanofibers are mainly made up by smaller aluminum oxide elements (i.e., AlO and AlO_2_ as shown in Figure 1a,b) at acidic conditions, such as pH 1 and pH 3, respectively. In more alkaline conditions, like pH 9 and pH 13, our results suggest that 2D nanoplates and 3D nanocubes contain more aluminum hydroxide units, like Al(OH) and Al(OH)_2_, as shown in Figure 1d,e.

## 3. Materials and Methods

### 3.1. SALVI Fabrication

**Figure 5:**
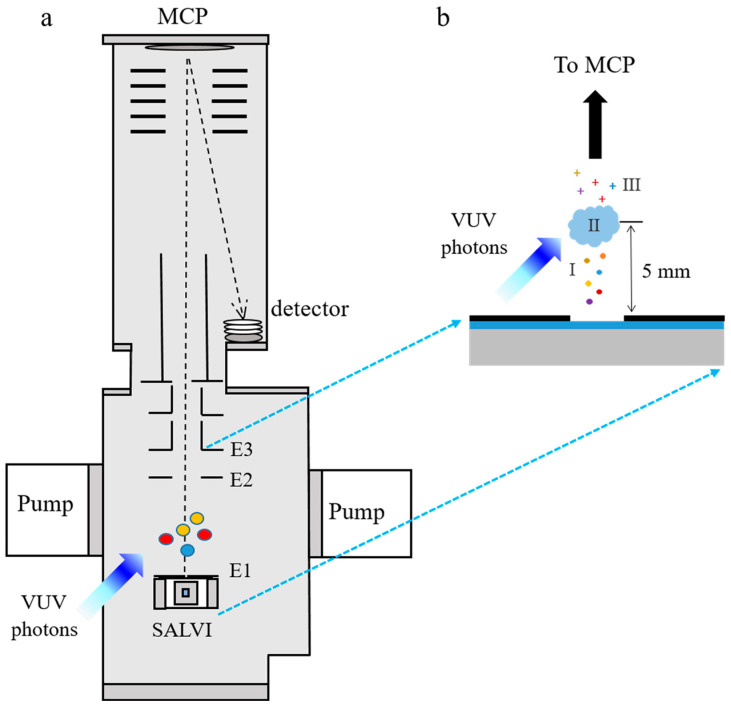
The experimental schematics of solvated boehmite particle study using VUV SPI-MS: (**a**) instrument diagram showing MCP with E1 as the bottom electrode plate, E2 the electrode held at 1000 V nominally, and E3 the electrode held at 0 V; and (**b**) the position of the VUV photon flux relative to evaporated molecules (I), photon beam (II), and molecules charged to positive ions (III) for detection, adapted from our recent papers [18,19,27].

The SALVI device is a portable and vacuum-compatible microfluidic reactor [21]. The rectangular microchannel is 200 μm (width) × 300 μm (depth) in a polydimethylsiloxane (PDMS) block. This channel is covered by a 100-nm thick SiN membrane (Norcada, Edmonton, AB, Canada, 1.5 × 1.5 mm^2^ in area) on a supporting 200-μm thick silicon frame of 7.5 × 7.5 mm^2^ in area to form the detection area. Figure 5 depicts the schematic of the SALVI device setup coupled with VUV SPI-MS at the Advanced Light Source (ALS) beamline 9.0.2. After microfluidics fabrication using soft lithography, two micropores (2 µm in diameter and 100 µm apart, Figure 5b) were milled on the center of the channel using the scanning electron microscopy-focused ion beam (SEM-FIB, ThermoFisher Helio Dual Beam Quanta, ThermoFisher, Waltham, MA, USA) to allow evaporation of molecules in vacuum and provide ions for ionization in high vacuum [18,19]. The chamber vacuum was kept on the order of 10^−7^ Torr during analysis. More details of SALVI integration to VUV SPI-MS are described in our recent studies [18,19].

### 3.2. Boehmite Sample Preparation

The boehmite stock solution of 1 mg/mL was prepared using 10 mg of AOH60 powder (AOH60 powder, Apyral, Nabaltec, Schwandorf, Germany) particle size 0.4 to 1.6 μm with D_50_ 1 μm) and deionized water (DI water) with resistance of 18.2 MΩ (Barnstead water purification system, Nanopure diamond, ThermoFisher, Waltham, MA, USA). The stock solution was then ultrasonicated for 5 min. The 10 μg/mL boehmite solution was made by diluting 1 mL boehmite stock solution into 99 mL DI water. Afterwards, a series of samples with different pH conditions of the 10 μg/mL boehmite (including pH 1, 3, 7, 9, and 13) were prepared. The pH of boehmite solution was adjusted by adding a suitable amount of hydrochloric acid (HCl) and sodium hydroxide (NaOH) prior to VUV SPI-MS analysis, and the solution pH can be regarded as constant over time. The solution pH was determined using a pH meter (Fisherbrand™ accumet™ AP110 Portable pH Meter Kit, ThermoFisher, Waltham, MA, USA). In order to reduce possible interference of PDMS signals in mass spectral analysis, each device was kept baking at 70 °C in a vacuum oven (ThermoFisher, Fisherbrand™ Isotemp™ Model 281A Vacuum Oven, ThermoFisher, Waltham, MA, USA) overnight prior to usage. The prepared boehmite solutions were then injected into the microchannel and sealed with PEEK fittings (Agilent Technologies, Santa Clara, CA, USA) for VUV SPI-MS analysis. The experimental schematic is depicted in Figure 5.

### 3.3. VUV SPI-MS Liquid Analysis

The SALVI device was attached to the bottom electrode plate of the microchannel plate (MCP) after the pH-adjusted boehmite solutions injected into the microchannel (Figure 5a). The VUV SPI-MS analysis was carried out by a three-meter VUV monochromator at the 9.0.2 Chemical Dynamics Beamline at the ALS of the Lawrence Berkeley National Laboratory [16]. The VUV photon flux was adjusted to about 5 mm above the SiN membrane by optimizing the height of the mass spectrometer before data collection [18,27,28]. After a ~20 min pump-down process, the chamber was maintained at the desirable vacuum 10^−7^ Torr during sample analysis. The molecules evaporated at a rate of about 10^8^ cm^−2^·s^−1^ (calculated by a custom code) from the two holes on the SiN membrane were ionized by the bright and tunable synchrotron VUV photon light (Figure 5a) [19,27]. Meanwhile, surface tension at the aqueous surface can hold the liquid in the micrometer-sized aperture along the microchannel and keep the vacuum stable. Recently, gamma-irradiation was found to facilitate the aggregation of boehmite particles in solution [29]. Nevertheless, as a “soft” ionization method, the VUV irradiation will not likely change the structure of evaporated molecules from the microchannel. Thus, the mass spectral results can provide more insights into the pH effect on boehmite dissolution in liquids. To optimize signals, the MCP voltage was tuned for detection of ions of interest. Ions were collected and detected by the ToF mass spectrometer. SPI-MS measurements were acquired for photon energies ranging from 8.0 to 12.6 eV for all boehmite samples. The energy step was 0.1 eV to obtain the PIE curve of each mass by integrating the peak intensities at each photon energy.

### 3.4. Data Analysis

Mass calibration of the VUV SPI-MS was performed by using known chemical compounds like water, oxygen, or rare gases [18]. During data acquisition, a continuous wave ion beam was converted to a pulsed one to obtain more photons [16]. The resulting mass resolution was not uniform over the mass range because higher relative energy is acquired by small molecules, which leads to narrower peaks. On the contrary, lower relative energy acquired by larger molecules can produce wider peaks. Thus, unit mass at the peak center was used for peak identification. The peak centers are slightly different at various photon energies for a particular species. However, the peak identifications based on the unit mass are consistent among different PIEs. For example, the ions detected at 12.5 eV, 12.0 eV, and 11.5 eV were consistent, as shown in Figure S2. All mass spectra were plotted and integrated in Origin^®^ 2018.

In this study, the AEs of key solvated boehmite ions were all smaller than 11.5 eV according to mass spectral analysis, as shown in Figure 3. However, identification of ions may be more difficult at lower IEs because the characteristic peaks have lower ion counts. Therefore, mass spectra at 12.5 eV were chosen for peak identification after evaluation of all spectra. The AE was determined to be the first point on the PIE curve where ion counts began to increase from the background signal. There was a slight difference in the total counts of different aqueous solutions of boehmite particles because of random surface topography and instrument efficiency. Thus, normalized mass spectral results were used to reduce uncertainties, as shown in Figures S1 and S2.

As discussed above, the mass of ions could not be absolutely determined because unit mass is used for peak identification. However, peak separation is not a problem since peak positions can be identified to two decimal places of accuracy. To improve the accuracy of identification, the key reactants/products were identified according to the following criteria. First, the magnitude of peaks was higher than their neighboring peaks and at least three times higher than the baselines. Second, product peaks reported in previous papers relevant to boehmite hydrogen and/or hydroxide behavior were included and searched in the spectra to verify their observations. Furthermore, three approaches were adopted to improve the likelihood and reliability of peak identification. First, peaks, except interference, such as system residuals from previous experiments and PDMS fragments, that have higher intensities than adjacent peaks were selected as alternative key products, reactants, or fragments. To minimize the interference from PDMS peaks in peak identification, all possible PDMS peaks (shown in Table S1) are smoothed (counts divided by 10) in the spectra. Second, the identities of peaks were proposed based on existing knowledge of boehmite solvation products [2,26,30]. Thirdly, peak identification was based on the AEs and PIE curves comparison with the reported values of chemicals coupled together with molecular mass determined from the spectral analysis. As to the observed species where AEs and/or structures that have not been found in previous studies, the new makeup and structure was deduced by considering the spectrum, PIE curve, and the most likely structures based on knowledge of the boehmite dissolution and the known literature (as shown in Table 1) [22,23].

## 4. Conclusions

In this study, we performed in situ VUV SPI-MS analysis of dissolved boehmite particles in different pH conditions in high vacuum. Liquid analysis in VUV SPI-MS was made possible by using the vacuum compatible SALVI microreactor. The dissolution pathways of boehmite at the solid–liquid, particularly in liquid water are more complex than previously conceived. Specifically, known dissolved products of boehmite, such as AlO^+^ and AlOOH^+^, are confirmed by their AEs determined from the PIE curves in addition to mass spectral analysis. Moreover, the AE values of newly found dissolution products have been reported, and their molecular structures reported with mass spectral analysis for the first time. Different types of dissolved ions were observed, and they show distinct intensities at different pH conditions. These new findings show that the solvation of boehmite depends on pH; and pH could indicate a change in the water microenvironment and affect the hydrogen bounding in aqueous solutions. Our results imply that in future studies on boehmite in liquid, such as temperature and ionic strength dependence, it is highly important to understand how boehmite dissolution could be impacted in the microenvironment in the existence of cluster ions in waste streams. We provide new insights into the pH dependence effect of boehmite; such fundamental understanding is important to design nuclear waste processing and environmental mitigation solutions. In future studies, quantum chemical calculations are desirable to determine the products more precisely by comparing the experimentally obtained AEs of ions with theoretical predications.

## Figures and Tables

**Table 1:** Identified key ions of dissolved boehmite particles at 12.5 eV in VUV SPI-MS in this work.

^1^ *m*/*z*^+^_obs_	^2^ *m*/*z*^+^_the_	Possible Identification	^3^ AEs (eV)
43	42.98	AlO^+^	10.8
60	59.99	AlOOH^+^	10.7
78	78.00	Al(OH)_3_^+^	10.8
97	96.94	Al(OH)_2_^+^…2H_2_O	11.2
106	105.99	(AlOH)_2_^+^…H_2_O	8.6
198	197.98	AlOOH…Al(OH)_2_…Al(OH)_3_^+^	10.3
313	313.02	[Al(OH)_3_]_4_H^+^	11.2
315	314.93	(AlOH)_4_…(AlOOH)_2_…H_3_O^+^	10.3
322	321.88	(AlO_2_)_2_…(Al_2_O_3_)_2_^+^	11.4
324	323.90	(AlOOH)_2_…(Al_2_O_3_)_2_^+^	11.2

^1^ *m*/*z*^+^_obs_: observed mass to charge ratio (*m*/*z*) obtained in this experiment; ^2^ *m*/*z*^+^_the_: theoretical *m*/*z*; ^3^ AEs: appearance energy values determined in this work.

## Data Availability

The datasets generated for this study can be found in the article/Supplementary Materials, further inquiries can be directed to the corresponding author.

## Supplementary Materials

The following supporting information can be downloaded at: https://www.mdpi.com/article/10.3390/ijms26157254/s1.
