# Supplementary material for: Probing the pH Effect on Boehmite Particles in Water Using Vacuum Ultraviolet Single-Photon Ionization Mass Spectrometry
Source: Int J Mol Sci. 2025 Jul 27;26(15):7254. doi: 10.3390/ijms26157254 (PMC12346584; doi:10.3390/ijms26157254)
Supplement: Supplementary file 1 [file ijms-26-07254-s001.zip › ijms-3701350-supplementary.pdf]

Table of Content

Supporting Figures ... 3

Figure S1a. VUV SPI-MS spectra of boehmite at pH 9 acquired at PIE of 8.5, 9.5, 10.5, 11.5 and 12.5 eV, respectively. ... 4

Figure S1b. VUV SPI-MS spectra of boehmite at pH 9 acquired at PIE of 8.5, 9.5, 10.5, 11.5 and 12.5 eV, respectively. ... 5

Figure S1c. VUV SPI-MS spectra of boehmite at pH 7 acquired at PIE of 8.5, 9.5, 10.5, 11.5 and 12.5 eV, respectively. ... 6

Figure S1d. VUV SPI-MS spectra of boehmite at pH 3 acquired at PIE of 8.5, 9.5, 10.5, 11.5 and 12.5 eV, respectively. ... 7

Figure S1e. VUV SPI-MS spectra of boehmite at pH 1 acquired at PIE of 8.5, 9.5, 10.5, 11.5 and 12.5 eV, respectively. ... 8

Figure S2a. Normalized VUV SPI-MS spectra of boehmite at 12.0 eV. ... 9

Figure S2b. Normalized VUV SPI-MS spectra of boehmite at 11.5 eV. ... 10

Supporting Table ... 11

Table S1. Summary of PDMS peaks observed in the positive ion mode. ... 11

Supporting Figures

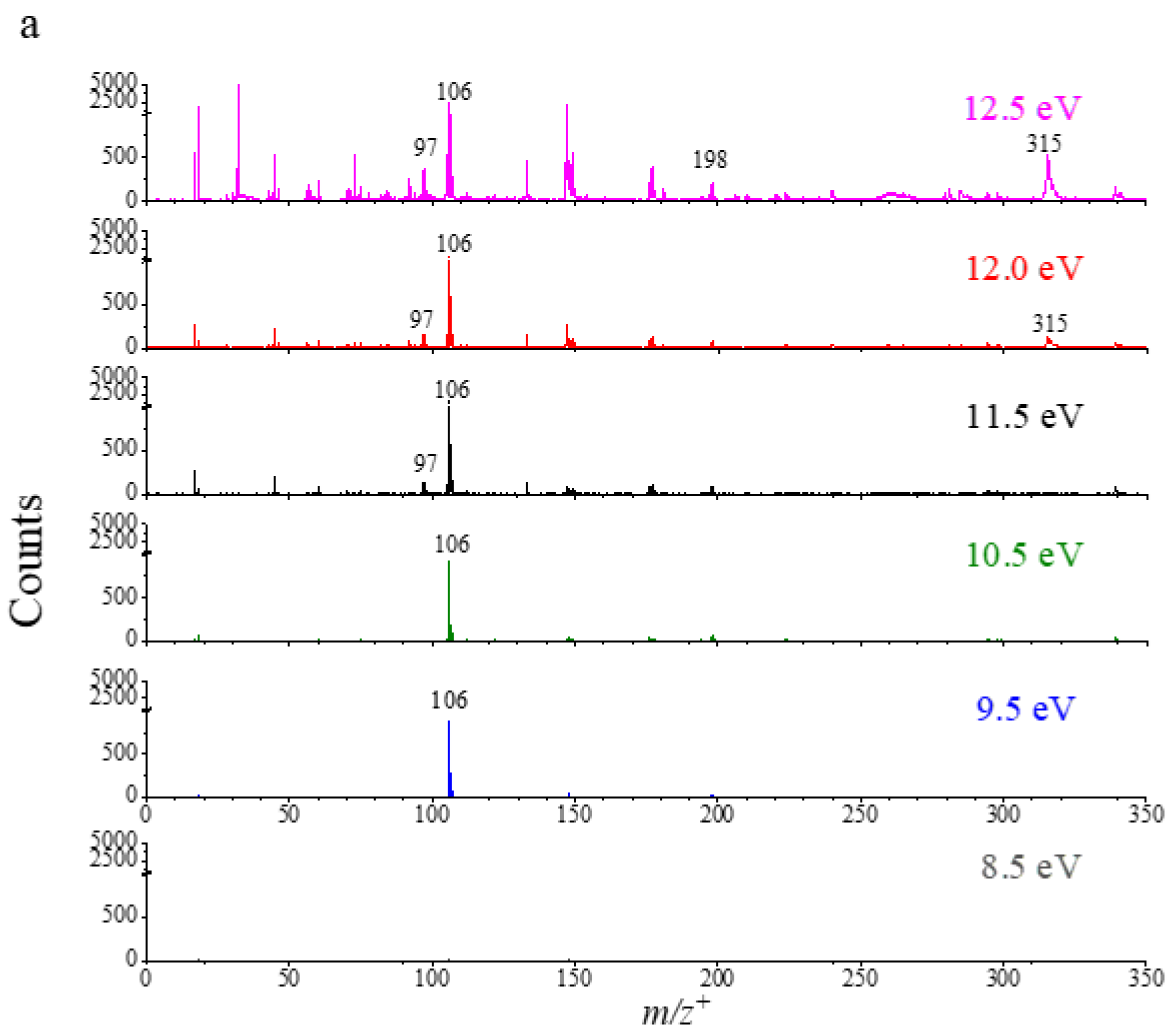

Figure S1a. VUV SPI-MS spectra of boehmite at pH 13 acquired at PIE of 8.5, 9.5, 10.5, 11.5 and 12.5 eV, respectively.

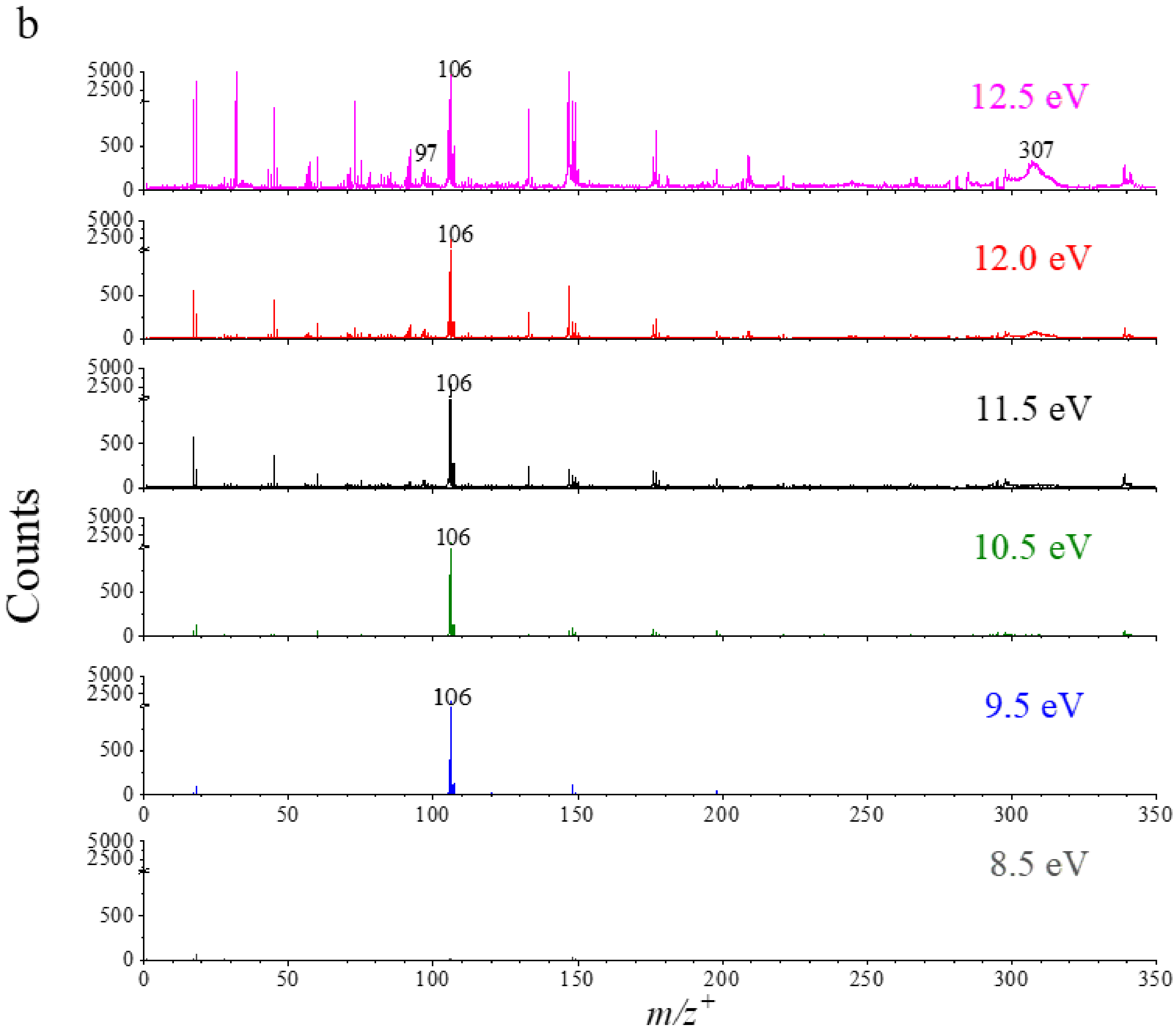

Figure S1b. VUV SPI-MS spectra of boehmite at pH 9 acquired at PIE of 8.5, 9.5, 10.5, 11.5 and 12.5 eV, respectively.

c

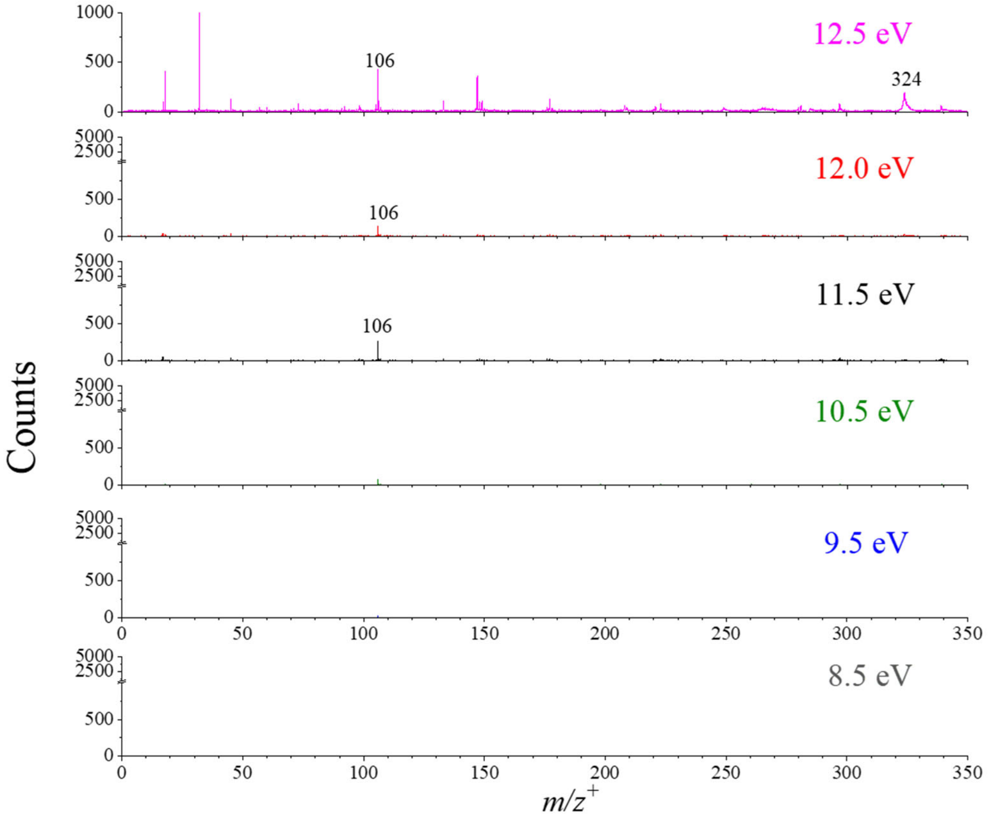

Figure S1c. VUV SPI-MS spectra of boehmite at pH 7 acquired at PIE of 8.5, 9.5, 10.5, 11.5 and 12.5 eV, respectively.

d

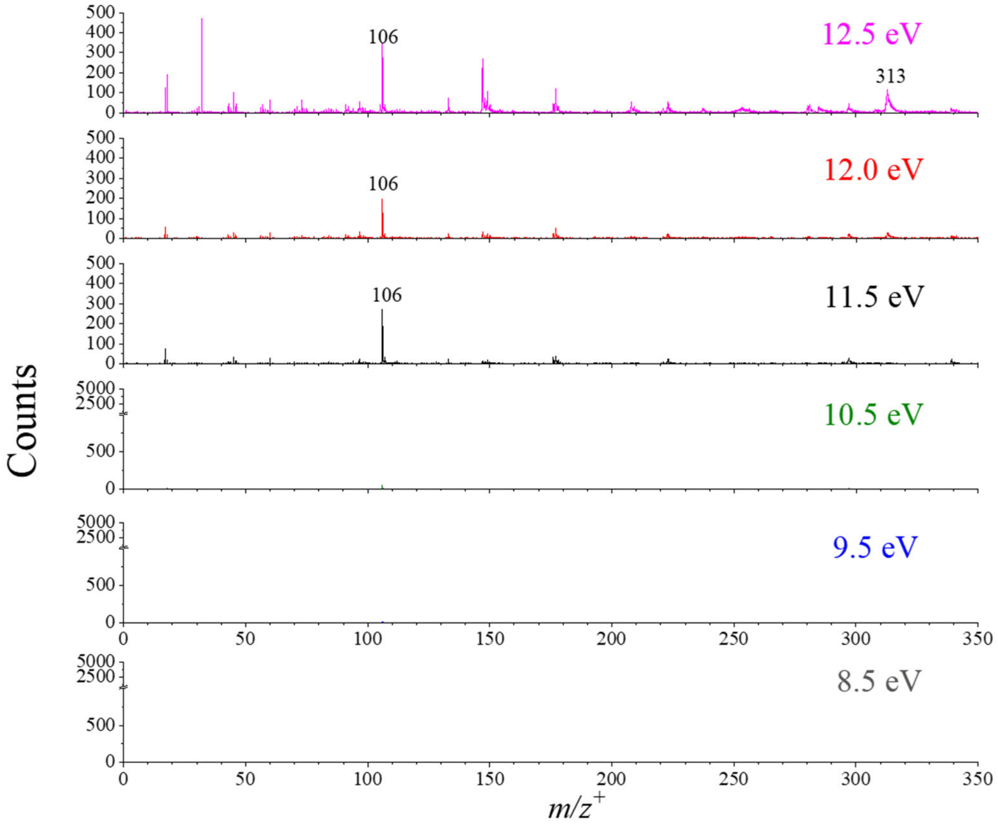

Figure S1d. VUV SPI-MS spectra of boehmite at pH 3 acquired at PIE of 8.5, 9.5, 10.5, 11.5 and 12.5 eV, respectively.

e

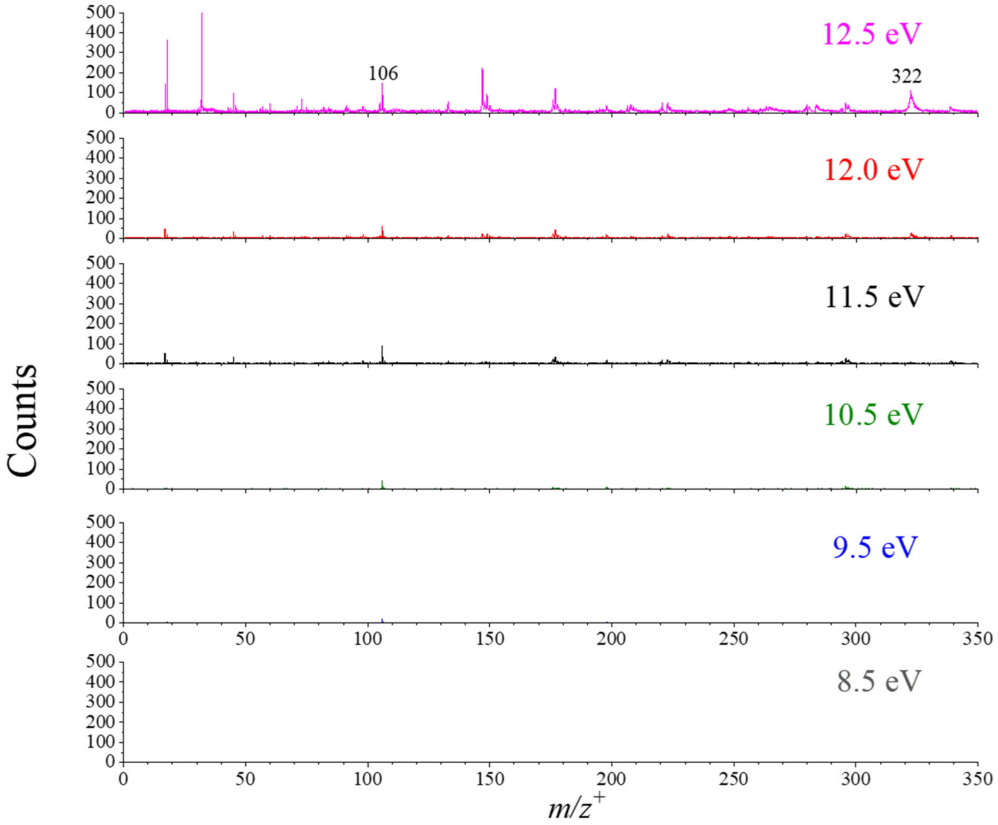

Figure S1e. VUV SPI-MS spectra of boehmite at pH 9 acquired at PIE of 8.5, 9.5, 10.5, 11.5 and 12.5 eV, respectively.

The peak centres are slightly different at different photon energies for a particular species. However, the peak identifications based on the unit mass are consistent among different energies. For example, the ions detected at 12.5 eV, 12.0 eV, and 11.5 eV were consistent, as shown in Figure S1. Also as shown in Figure S1, the species may be vague at lower IEs because the characteristic peaks are generated by fewer ions. Therefore, mass spectra at 12.5 eV were chosen for peak identification.

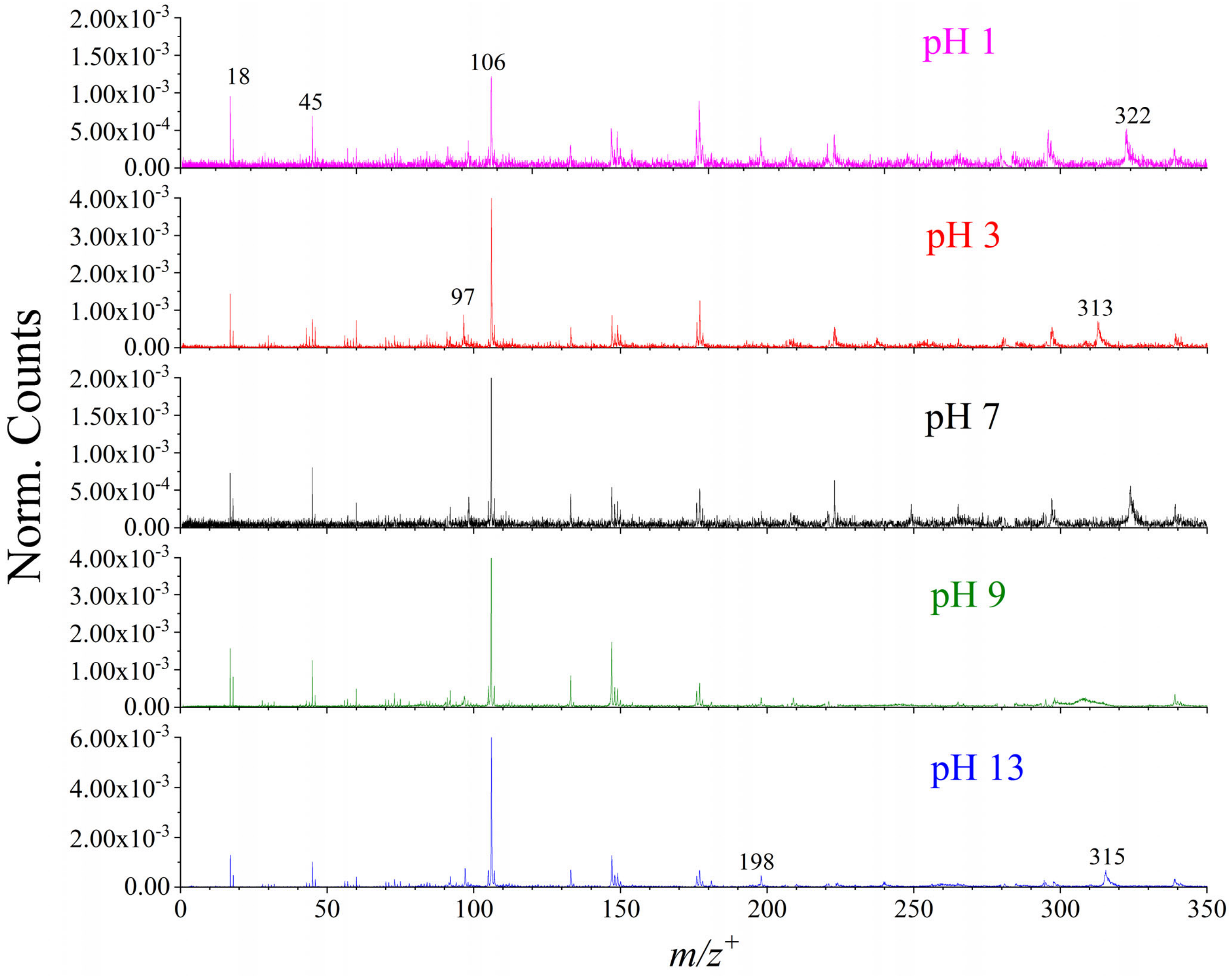

Figure S2a. Normalized VUV SPI-MS spectra of boehmite at 12.0 eV.

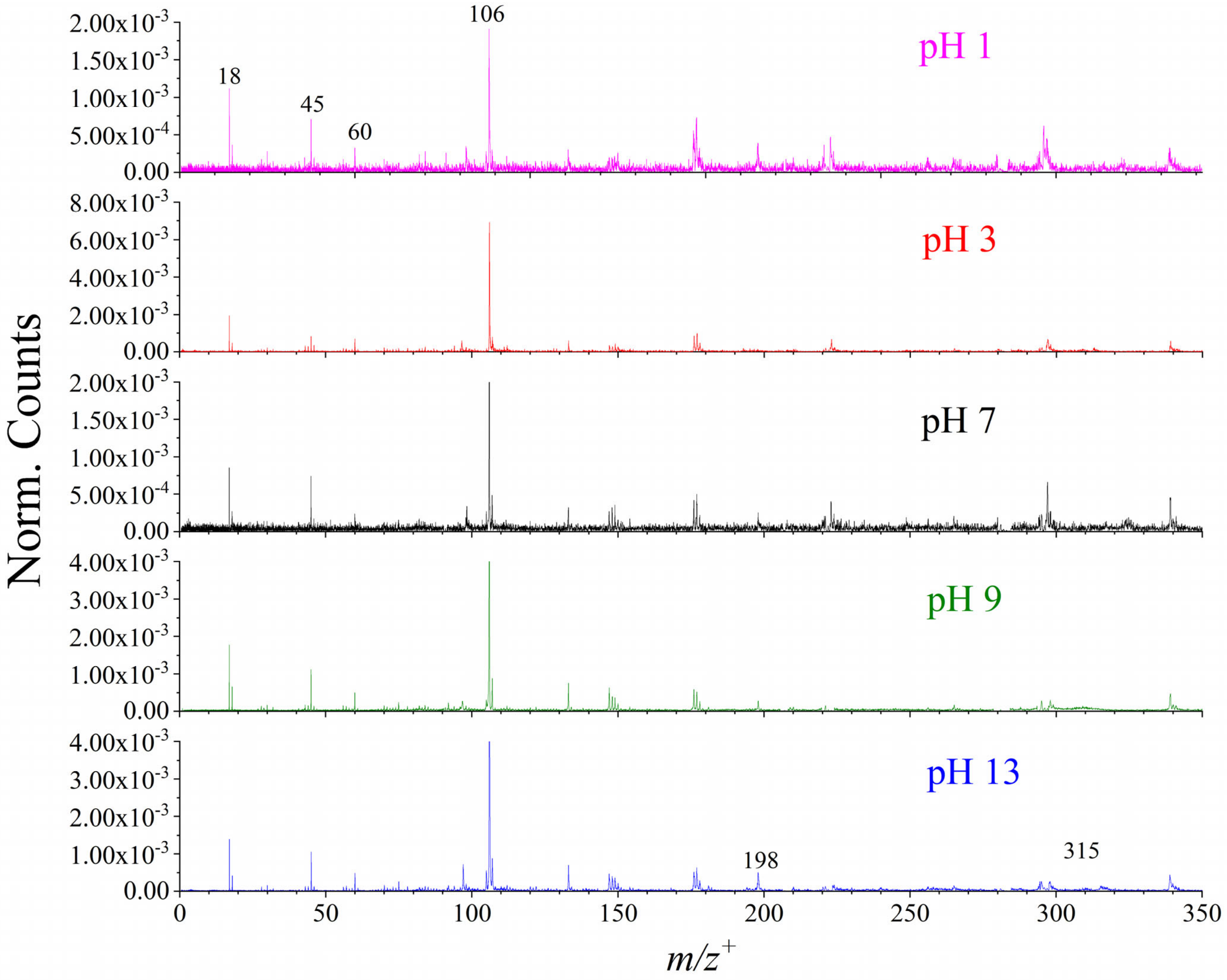

Figure S2b. Normalized VUV SPI-MS spectra of boehmite at 11.5 eV.

Figure S2 shows the normalized VUV SPI-MS spectra at two different PIE energies. The peaks included boehmite oxides and hydroxides like *m/z* 43 (AlO^+), *m/z* 60 ($AlOOH^+$), *m/z* 78 ($Al(OH)_3{}^+$), and *m/z* 97 (Al_3O^+) . Another kind of products are cluster ions, i.e. *m/z* 106 $(AlOH)_2{}^+ \ldots H_2O$, *m/z* 198 $AlOOH \ldots Al(OH)_2 \ldots Al(OH)_3{}^+$, *m/z* 313 $[Al(OH)_3]_4H^+$, *m/z* 315 $(AlOH)_4 \ldots (AlOOH)_2 \ldots H_3O^+$, *m/z* 322 $(AlO_2)_2 \ldots (Al_2O_3)_2{}^+$ and *m/z* 324 $(AlOOH)_2 \ldots (Al_2O_3)_2{}^+$.

Supporting Table

Table S1. Summary of PDMS peaks observed in the positive ion mode.

[1] m/z^+_{obs}	Possible identification
133	$C_3H_9O_2Si_2$
147	$C_5H_{15}OSi_2$
221	$C_7H_{21}O_2Si_3$
239	$C_4H_{15}O_4Si_4$
281	$C_9H_{27}O_5Si_5$
295	$C_9H_{27}O_3Si_4$
297	$C_8H_{25}O_4Si_4$
133	$C_3H_9O_2Si_2$
147	$C_5H_{15}OSi_2$
221	$C_7H_{21}O_2Si_3$

[1] m/z^+_{obs}: observed mass to charge ratio (m/z) obtained in this experiment,

Interferences that may come from PDMS off-gassing in vacuum were summarized in Table S1.
